# Retraction: Macrophage-derived exosomes mediate osteosarcoma cell behavior by activating AKT signaling

**DOI:** 10.1039/d1ra90043a

**Published:** 2021-01-28

**Authors:** Laura Fisher

**Affiliations:** Royal Society of Chemistry Thomas Graham House, Science Park, Milton Road Cambridge CB4 0WF UK advances-rsc@rsc.org

## Abstract

Retraction of ‘Macrophage-derived exosomes mediate osteosarcoma cell behavior by activating AKT signaling’ by Bin Yan *et al.*, *RSC Adv.*, 2020, **10**, 5032–5039, DOI: 10.1039/C9RA07332A.

The Royal Society of Chemistry hereby wholly retracts this *RSC Advances* article due to concerns with the reliability of the data. The images in the article were screened by an image integrity expert. The western blots in the article all have very low contrast and may not be genuine. The authors were asked to provide the raw data for the article, but they stated that they were unable to locate the primary raw data for the western blots in Fig. 1 and 5. Therefore, the authors are unable to provide raw data that can be used to validate the published data.

Given the significance of the concerns about the validity of the data, and the lack of raw data, the findings presented in this paper are not reliable.

The authors have been informed and do not agree with the retraction.

Signed: Laura Fisher, Executive Editor, *RSC Advances*

Date: 15^th^ January 2021

## Supplementary Material

